# Recent Developments in Polyphenol Applications on Human Health: A Review with Current Knowledge

**DOI:** 10.3390/plants12061217

**Published:** 2023-03-07

**Authors:** Nikheel Bhojraj Rathod, Nariman Elabed, Sneh Punia, Fatih Ozogul, Se-Kwon Kim, João Miguel Rocha

**Affiliations:** 1 Post-Graduate Institute of Post-Harvest Technology and Management, Dr. Balasaheb Sawant Konkan Krishi Vidyapeeth, Roha 402 116, India; 2 Laboratory of Protein Engineering and Bioactive Molecules (LIP-MB), National Institute of Applied Sciences and Technology (INSAT), University of Carthage, BP 77-1054 Amilcar, Carthage 1054, Tunisia; 3 Department of Food, Nutrition and Packaging Sciences, Clemoson University, Clemosn, SC 29634, USA; 4 Department of Seafood Processing Technology, Faculty of Fisheries, Cukurova University, 01330 Adana, Turkey; 5 Biotechnology Research and Application Center, Cukurova University, 01330 Adana, Turkey; 6 Department of Marine Science & Convergence Engineering, College of Science & Technology, Hanyang University, ERICA Campus, Ansan 11558, Republic of Korea; 7 LEPABE—Laboratory for Process Engineering, Environment, Biotechnology and Energy, Faculty of Engineering, University of Porto, Rua Dr. Roberto Frias, 4200-465 Porto, Portugal; 8 ALiCE—Associate Laboratory in Chemical Engineering, Faculty of Engineering, University of Porto, Rua Dr. Roberto Frias, 4200-465 Porto, Portugal

**Keywords:** polyphenols, flavonoids, bioactivity, anti SARS-CoV-2, human health

## Abstract

Polyphenol has been used in treatment for some health disorders due to their diverse health promoting properties. These compounds can reduce the impacts of oxidation on the human body, prevent the organs and cell structure against deterioration and protect their functional integrity. The health promoting abilities are attributed to their high bioactivity imparting them high antioxidative, antihypertensive, immunomodulatory, antimicrobial, and antiviral activity, as well as anticancer properties. The application of polyphenols such as flavonoids, catechin, tannins, and phenolic acids in the food industry as bio-preservative substances for foods and beverages can exert a superb activity on the inhibition of oxidative stress via different types of mechanisms. In this review, the detailed classification of polyphenolic compunds and their important bioactivity with special focus on human health are addressed. Additionally, their ability to inhibit SARS-CoV-2 could be used as alternative therapy to treat COVID patients. Inclusions of polyphenolic compounds in various foods have demonstrated their ability to extend shelf life and they positive impacts on human health (antioxidative, antihypertensive, immunomodulatory, antimicrobial, anticancer). Additionally, their ability to inhibit the SARS-CoV-2 virus has been reported. Considering their natural occurrence and GRAS status they are highly recommended in food.

## 1. Introduction

Polyphenols are naturally occurring secondary bioactive compounds derived from plant sources, which show a wide range of bioactivity helping in promoting good health [1,2]. They are basically phenolic rings with attached functional groups. Application of polyphenols has received great attention in all segments from food processing to preservation and the pharmaceutical industry [3,4,5,6,7]. Previous studies have also shown application of several phenols in the formulation of traditional medicine [8,9].

Oxidative stress, hypertension, lowered immunity, microbial infections, and antimicrobial resistance have been reported to cause a large number of deaths globally [10]. The diverse bioactivity possessed by phenols is attributed to their structure (ring) that makes them capable of helping in the treatment of various diseases and disorders [11]. The COVID-19 pandemic has caused the full or partial closure of many countries due to the rapid spread of the virus and higher mortality rate, hampering businesses with an increase in work from home [12]. Polyphenols from natural resources are known to enhance immunity by modulating defence mechanisms [13,14].

Considering the wide availability of the polyphenols, their detailed classification, types, and sources are described. Recent developments relating to bioactivity of polyphenols as well as their antiviral activity against SARS-CoV-2 are also reviewed. Future prospects proposing the direction requiring research are also addressed.

## 2. Polyphenols

### 2.1. Classification

**Figure 1:**
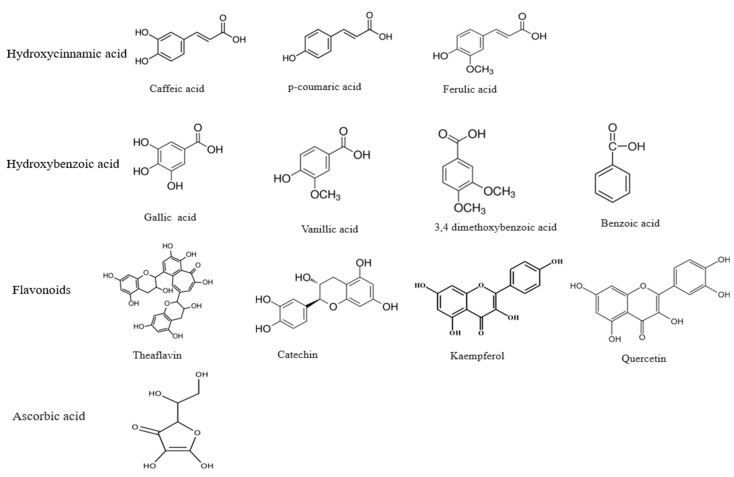
Some important polyphenolic compounds.

Dietary polyphenols comprise a broad category of natural compounds in the kingdom Plantae with two phenyl rings and one or more hydroxyl (OH) groups [15]. Approximately 8000 polyphenolic compounds are currently known, and more than 4000 belong to flavonoids only [16]. Polyphenols are a heterogeneous group of phenolic compounds [17] with two major classes: flavonoids and phenolic acids. Flavonoids are coloured compounds subdivided into flavones, flavanols, flavanones, flavonols, and isoflavones [18], whereas phenolic acids have two subgroups, namely hydroxycinnamic and hydroxybenzoic acids [19]. They are found in either a non-conjugated (aglycone) or conjugated form with glucose, organic acid, carboxylic acid, amines, lipids, etc. [18,20]. The structures of some important polyphenols are given in Figure 1.

### 2.2. Types

**Figure 2:**
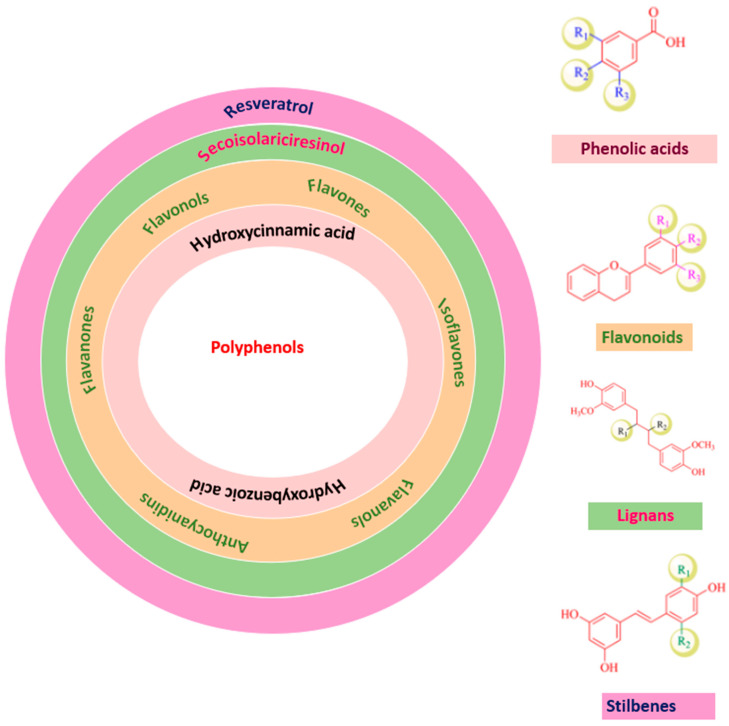
Polyphenols classification.

Phenolic compounds have many subclasses based on phenol units in the molecule, substituent groups, and the bond type between phenol units (Figure 2). Depending on their structure variation, polyphenols are categorized into phenolic acids, phenolic aldehyde (vanillin, salicylaldehyde, syringaldehyde, etc.), flavonoids, iso-flavonoids, tannins (hydrolyzable and condensed tannins), lignans, and lignins [21,22].

#### 2.2.1. Phenolic Acids

Phenolics are categorized as non-flavonoid polyphenols with several OH groups on aromatic rings. Two distinguishing parent skeletons of phenolic acids include benzoic acid containing seven C atoms (C6-C1), and cinnamic acid containing nine carbon atoms (C3-C6) [23]. Hydroxybenzoic acids that contribute to the human diet are rare, thus, not suggested to play a role in human health [19]. Benzoic acid derivatives are p-hydroxybenzoic acid, protocatechuic, salicylic, gallic, and ellagic acid, as well as cinnamic acid derivatives include p-coumaric, caffeic, and ferulic acid [24]. Phenolic acids are essential as human dietary components with tremendous health benefits, including antioxidative, anti-inflammatory, immunoregulatory, anti-allergic, anti-atherogenic, anti-microbial, cardioprotection, anti-cancer, and antidiabetic potential [25].

#### 2.2.2. Flavonoids

Flavonoids are ubiquitous polyphenols that contribute to colourful pigments in fruits, vegetables, herbs, spices, and medicinal plants [26]. They have 15 carbons and two aromatic rings joined by a three-carbon bridge. Depending on the C-ring difference, they are further divided into flavones, flavanones, isoflavones, flavonols, flavan-3-ols, and anthocyanidins [27]. Each subgroup has some differences due to the pattern and degree of hydroxylation, prenylation, glycosylation, or methoxylation. Some examples of flavonoids are quercetin, catechin, naringenin, cyanidin-glycoside, and daidzein [28,29]. They exist as free aglycones and glycosidic conjugates [30,31] as well as various modified forms [32].

##### Flavone and Flavonols

Flavones comprise a three-ring skeleton with three functional groups; a C4 ketone, a conjugated C2-C3 double bond, and various numbers depending on the flavone of hydroxyl groups [33]. Compared to flavonols, flavones lack an OH group at the three-position. Major flavonols are quercetin, kaempferol, myricetin, and isorhamnetin. The most abundant flavones in plants are luteolin and apigenin [34]. Approximately 450 types of flavonol aglycones have been reported, although quercetin, kaempferol, myricetin, and isorhamnetin are commonly found in fruits [35].

##### Isoflavones

Isoflavones and their derivatives are eco physiologically active secondary metabolites derived from the phenylpropanoid pathway [36]. They are yellow-pigmented compounds and found in plants mostly as biologically inactive glycosides: 7-*O*-β-D-glycosides, 6″-*O*-acetyl-7-*O*-β-D-glucosides, and 6″-*O*-malonyl-7-*O*-β-D-glycosides [37]. Isoflavones exist as phytoestrogens due to their binding affinity for estrogens receptors. Isoflavones differ from other flavonoids in the position of benzene ring B in C3 [38]. Dietary isoflavone has been a good way to study its health benefits, i.e., healthy gut, osteoporosis prevention, anti-inflammatory, anti-cancer, anti-obesity, and anti-diabetic potential [39].

##### Flavanones

Flavanones are a therapeutically important flavonoids class in all citrus fruits, including oranges, lime, lemons, grapefruit, and grapes [40]. Hesperidin, naringenin, and eriodictyol are some flavanones [41]. Moreover, they are widely distributed in 42 larger plant families, including Compositae, Leguminosae, and Rutaceae. The pharmacological potential of flavanones includes free radical scavenger, anti-inflammatory, anticancer, cardiovascular, and antiviral [42].

##### Anthocyanins

They are naturally occurring pigmented components and the most important subclass of flavonoids [43]. They are glycosylated polyhydroxy and poly methoxy derivatives of two phenylbenzopyrylium (flavylium) salts. Pelargonidin, cyanidin, peonidin, delphinidin, petunidin, and malvidin are some common anthocyanins [44]. They occur predominantly in the outermost layers of berries (raspberries, cranberries, strawberries, blueberries, bilberries, blackberries), black currants, and red and merlot grapes [41]. Anthocyanins can scavenge free radicals by two hypothesized pathways; the first pathway is the attack hydroxyl group/s of the B-ring. The next is the attack of oxonium ion on the C-ring. Some of them are considered among the strongest antioxidants by adopting both pathways [43,45,46]. Additionally, they have potential in food industries as natural dyes and replacers of synthetic dyes [47].

#### 2.2.3. Stilbenes

Another non-flavonoid class, stilbenes, contains two phenyl moieties attached by a 2-C methylene bridge. They contain two aromatic rings, A and B, and exist in isomeric (cis and trans), free and glycosylated forms. At m-position, ring A carries two hydroxyl groups, whereas many positions in ring B are substituted by hydroxy and methoxy groups [48]. Resveratrol (3,5,4′-trihydroxystilbene) is commonly stilbenes, which is produced by berries and grapes as well as nuts [49].

#### 2.2.4. Lignans and Lignin

Two C6-C3 units linked together between positions 8 and 8′ are called lignan, another non-flavonoid compound. The positions of lignan C9 and C9′ are substituted in different patterns, and, thus, they have different structural forms. They are divided into subgroups such as furan, dibenzylbutane, and aryltetralin [48]. Lignans are mainly free form in pulses, seeds, and vegetable oils as the glycosylated structures are not abundant. Lignin is an aromatic biopolymer formed by phenolic oxidative coupling of p-hydroxycinnamoyl alcohol monomers by peroxidase enzymes [50]. The most important alcohols are 4-hydroxycinnamoyl, coniferyl, and sinapoyl.

#### 2.2.5. Tannins

Tannins are a naturally occurring heterogeneous group of water-soluble high molecular weight phenolics. They are subdivided into hydrolyzable and condensed tannins. Hydrolyzable tannins are further categorized into gallotannins (hydrolysis yields sugar and gallic acid) and ellagitannins (hydrolysis yields sugar, gallic and ellagic acid [51]. They exhibit free radical scavenging potential (due to hydroxyl groups), which increases as the number of galloyl groups and molecular weight increase, and in the presence of an*o*-dihydroxy structure [50].

Recently, phlorotannins, types of tannin found in the sub-cellular structure of brown algae (accounting 25%) are gaining interest in food, feed, and drug industries [52]. They are polyphenolic compounds formed by polymerization of phloroglucinol (1,3,5-trihydroxybenzene) with a wide range of molecular sizes of between 126 and 650,000 Da. Based on linkage, phlorotannins are divided into four categories, including fuhalols (ether linkage), phlorethols (ether linkage), fucophloroethols (ether and phenyl linkage), and eckols (dibenzodioxin linkage) [53]. Phlorotannins have shown a great deal of promise as functional compounds with a number of bioactivities including anti-cancer, anti-diabetic, anti-inflammatory, anti-microbial, and anti-hypertensive [54]. Additionally, these phenolic compounds have significant scavenging potential against superoxide and free radicals so they could be used as antioxidants in the food industries. Beside these functionalities, they also serve as pancreatic lipase inhibitors, which have the potential to control dietary fat digestion. Hence, it can be used for weight control purposes [55].

### 2.3. Sources

There is growing interest in identifying and exploring polyphenolic sources to prepare functional beverages, extract polyphenolic compounds to fortify functional foods and beverages, or elaborate dietary supplements [56]. They have antioxidant properties that complement the functionality of vitamins and enzymes as a defence against oxidative stress caused by excessive ROS production [23]. Major polyphenolic sources are tabulated in Table 1.

## 3. Impacts of Polyphenols on Human Health

The polyphenols are known as secondary metabolites widely present in the plant kingdom. Polyphenols have important health-promoting impacts according to their antioxidant, antimicrobial, immunomodulatory, antihypertensive, anticancer, and anti-inflammatory properties.

### 3.1. Antioxidant Activity

Polyphenols are considered among the phytochemical compounds, which have been recognized as bioactive and functional compounds. They have attracted tremendous attention from food scientists, nutritionists, and consumers owing to their considerable healthful benefits. It has been demonstrated by recent research studies that dietary polyphenols have several important biological properties such as antioxidant activity, which has received great interest. The antioxidant activity of some phenolic compounds is summarized in Table 2.

Generally, oxidative stress appears when there is weak antioxidant protection, or high production of reactive oxygen species (ROS), including hydroxyl radicals (•OH), superoxide anion radicals (O2^•−^), and hydrogen peroxide (H_2_O_2_), during cellular respiration and other different metabolic pathways. Such processes cause chemical reactions and damage to cells, tissues, and several biomolecules, including DNA, proteins, lipids, and carbohydrates [3,57,58]. Consequently, oxidative damage leads to many human chronic diseases including atherosclerosis, inflammation, cancers, diabetes, heart attack, arthritis, liver injury, neurological disorders, cataractogenesis, and retinal damage as well as other several degenerative diseases [3,57,59].

**Table 1:** Sources of polyphenols.

Polyphenols	Food Source	Content	Unit	Reference
Total phenols	Berry	85.80–1097.44	µg GAE/mL)	[56]
	Oat	7.6–16.8	mg GAE/g	[60]
	Barley	2890–3922	μg FAE/g	[61]
	Wheat	1650–2095	µg GAE/g	[62]
	Wheat	160	µmol FAE/100 g	[63]
	Rice	20–47.84	mg GAE/g	[64]
	Rye	0.984–3.369	mg GAE/g	[65]
	Corn	451–4899	mg/kg DW	[66]
	Pearl millet	2394–3137	µg GAE/g	[67]
	Broccoli	40–100	mg/L	[68]
	Kiwi	600–1000	mg/L	[68]
	Black carrot	311.5	mg/100 g	[69]
	Grape	9.95–146.32	mg/100 g	[70]
	Tea	152–243	mg GAE/g	[71]
	Tomato	1422–1564	mg/100 g	[72]
	Onion	1221–1483	mg/100 g	[72]
	Apple	905–1030	mg/100 g	[72]
Phenolic acids				
Caffeic acid	Grape	9–138.21	mg/100 g	[70]
p-coumaric acid	Corn flour	18.69	μg/g	[73]
	Rye	0.343–1.280	mg/kg	[65]
	Barley	14.61–583.54	μg/g	[74]
	Finger millet	1.81	μg/g	[75]
Ferulic acid	Corn flour	155.69	μg/g	[73]
	Wheat	25.40	μg/g	[76]
	Barley	5.61–13.88	μg/g	[74]
	Rye	1.903–6.227	mg/kg	[65]
	Pearl millet	160	μg/g	[77]
	Coffee	0.09–0.14	g/kg	[78]
	Broccoli	1.95	μg/g	[79]
	Banana	0.49–0.53	g/kg	[80]
	Mango	0.75	g/kg	[80]
Catechins	Rice	0.26–3.98	mg/100 g	[81]
	Tea	3145.04–13,986.41	mg/100 g	[82]
Theaflavins	Tea	4.07–1109.78	mg/100 g	[82]
Gallic acid	Rice	5.43	mg/100 g	[81]
	Pearl millet	120	μg/g	[77]
	Black rice	1.4	mg/g	[83]
	Barley bran	405.5	μg/g	[84]
Vanillic acid	Rye	1.086–3.130	mg/kg	[65]
Benzoic acid	Barley	8.81–528.56	μg/g	[74]
3,4 dimethoxybenzoic acid	Barley	18.51–110.85	μg/g	[74]
Ascorbic acid	Barley bran	20.44	μg/g	[84]
	Pearl millet	320	μg/g	[77]
	Dried litchi peel	225.98	mg/100 g	[85]
Total flavonoids	Berry	17.45–67.37	µg RE/mL	[56]
	Wheat	75–121	µg CE/g	[62]
	Barley	1968–2198	μg FAE/g	[61]
	Rice	3.35- 7.14	µg RE/g	[64]
	Rye	0.042–0.203.36	mg QE/g	[65]
	Pearl millet	1721–2484	µg CE/g)	[67]
	Grape	20.15–46.27	mg/100 g	[70]
Flavonoids				
Kaempferol	Grape	15.31–43.80	mg/100 g	[70]
	Corn flour	14.58	μg/g	[73]
	Broccoli	3.42	μg/g	[79]
Quercetin	Oat	12.2–51.6	μg/g	[86]
	Buckwheat	3.1–6.71	μg/g	[86]
Anthocyanins	Berry	8.08–21.28	µgC3GE/mL	[56]
	Black carrot	837.9	mg/100 g	[87]
	Rice	0.26–256.5	mg/100 g	[81]
	Corn	307–321	mg/kg DW	[66]
	Black wheat	185.8	mg/kg	[48]
	Pigmented maize	23 to 252	μg/g	[88]

In this regard, the use of natural antioxidant compounds derived from plant-based food, such as dietary plant polyphenols, can contribute to the defense of the human body against oxidative stress and its adverse effects. These phytochemical compounds play an important protective role against different diseases and they are considered among the most significant natural antioxidants used in the human diet [89].

The phenolic compounds are considered active antioxidant compounds that can treat and prevent several degenerative diseases through ROS scavenging capacity and the regulation of the activity of various oxidase species in the organism [58]. It has been reported that the ingestion of dietary polyphenols as natural antioxidants can prevent mortality and morbidity caused by the degenerative diseases as well as contribute to the prevention of lipid oxidation in foods, such as fish and seafood [2,90]. Moreover, previous studies have demonstrated that the consumption of some food products enriched with oxidized lipids lead to an increase in the concentration of toxic malonic aldehyde and hydroperoxides in the digestive system, particularly in the stomach. However, the consumption of food products containing a considerable quantity of polyphenol can decrease or inhibit the accumulation of such toxic substances. 

The polyphenols are characterised by their capacity to convert the relative free radical products to other non-reactive species in a more stable way [91]. They have also been demonstrated to have the capacity to regulate redox-dependent cellular signalling in living organisms [92]. The considerable antioxidant activity of phenolic compounds is due to the structure of phenolic hydroxyl in which the electrons possess a conjugation impact and the capacity of the hydrogen ion’s binding is reduced [58]. Consequently, there is a neutralization of the free radicals and reactive oxygen species (ROS) [58]. Actually, the antioxidant properties of these bioactive compounds are extremely related to the position and number of the phenolic hydroxyl groups [93].

Therefore, the phenolic compounds can eliminate the production of free radicals. Besides, these phytochemical compounds have a direct radical scavenger activity for the chain of reactions of lipid peroxidation (chain breakers). Through this process, there is a transfer of an electron to the free radicals and then the radicals become more stable by their neutralisation [23]. Thereby, the chain reaction will be stopped [23].

Besides, the polyphenols compounds have also been characterised by their metal chelating activity, which has a considerable role in the protection against the deterioration of DNA in the living cells [94]. The antioxidant mechanism of the phenolic compounds occurs by their ability to entrap the metal ions (for instance Fe^2+^ and Cu^2+^), which can be involved in the Fenton reaction in the existence of hydrogen peroxide. Thus, these bioactive compounds reduce and avoid the transition of metal chelation and then can prevent the oxidation by the inhibition of the production of free radicals. Consequently, the polyphenols can obtain stable compounds by their antioxidant activity [94,95]. 

In addition, in accordance with previous in vitro research studies, it has been demonstrated that these bioactive compounds are characterised by their cancer-preventing ability by the inhibition of the accumulation of reactive oxygen species (ROS) in human organisms [58]. Moreover, previously in-vivo research studies have mentioned that these phytochemical substances can raise the level of superoxide dismutase (SOD), the glutathione peroxidase (GSH-Px), and the serum catalase (CAT), and can then inhibit the generation of malondialdehyde (MDA) [58]. Thus, the polyphenols can lead to the regulation of the oxidoreductase systems, and, hence, they can enhance the antioxidant capacity of the organisms [58].

**Table 2:** Antioxidant activity of some phenolic compounds.

Sources	Compounds	Concentrations	Assays	Main Findings	Reference
*Vaccinium corymbosum* L. (Blueberry fruits)	Anthocyanins Phenolic acids Flavonols	200 g/day	Ferric reducing antioxidant potential (FRAP). Total radical-trapping antioxidant parameter (TRAP). Total antioxidant capacity (TAC) assays.	The ingestion of blueberry fruits enriched with phenolic compounds contributes to the inducing of an important increase of endogenous plasmatic antioxidant protection.	[96]
*Thymus lotocephalus*	Caffeic acids Rosmarinic acids Apigenin Luteolin	- 2% - 94.7% - 1.7% - 1.6%	Trolox equivalent antioxidant capacity (TEAC) assay. Oxygen radical absorbance capacity (ORAC) assay. Fe^2+^ chelation assay. Lipid peroxidation assay.	The phenolic compounds found in *Thymus lotocephalus* are characterized by efficient antioxidant activities. The use of different antioxidant assays (ORAC and TEAC assays) can neutralize free radicals by leading to the production of complexes with Fe^2+^ and then the protection of mousse brains against lipid peroxidation induced by Fe^2+^.	[97]
*Artemisia campestris* L.	Condensed tannin Other phenolic compounds	- 0.48 mg EC/gDW - 36.05 mg GAE/g DW	DPPH radical scavenging activity. Total antioxidant capacity by phosphomolybdenum.	The *Artemisia campestris* enriched with phenolic compounds have demonstrated their excellent antioxidant activity, with radical-scavenging activity (85.48%). Further, the polyphenol compounds exhibited a strong total antioxidant capacity (55.75 mg AAE/g DW).	[91]
*Thymelaea hirsuta* L.	Condensed tannin Other phenolic compounds	- 9.45 mg EC/gDW - 44.23 mg GAE/g DW	DPPH radical scavenging activity. Total antioxidant capacity by phosphomolybdenum.	The phenolic compounds and condensed tannin found in the plant of *Thymelaea hirsuta* exhibited an important antioxidant activity, which was demonstrated by their highest DPPH radical-scavenging activity (85.8%) and their excellent total antioxidant capacity (57.54 mg AAE/g DW).	[91]
*Ipomoea batatas* [L.] Lam (leaves harvested at BBCH stage 51 of development).	Phenolic acids: ✓ chlorogenic acid; ✓ neochlorogenic acid; ✓ caffeoylquinic acid. Flavonoids: ✓ Quercetin	5026.8 mg/100 g^−1^ DM	Ferric-Reducing/antioxidant power assay (FRAP). ABTS assay. DPPH assay.	The results of this study have demonstrated that important correlations existed between the level of polyphenol compounds and antioxidant properties determined by means of ABTS, DPPH, and FRAP assays.	[98]
*Ugni molinae*	Tannins Flavonoids Phenolic acids	10 mg/mL	DPPH assay. TBARS assay. TEAC-CUPRAC	The results of this study reported that the plant extract enriched with phenolic compounds was characterized by a considerable in vitro antioxidant activity via the DPPH assay. The consumption of leaves of *Ugni molinae* contribute to the decrease of TBARS and the increase of plasma antioxidant capacity (TEAC-CUPRAC).	[99]

However, the flavonoids represent a large class of low-molecular weight polyphenol compounds. This group can inhibit the nitric oxide synthase, which is responsible for the production of nitric oxide components. These latter can be considered as free radicals or react with other free radicals and then produce peroxynitrite species [95]. Furthermore, the flavonoids compounds, including luteolin and quercetin, can inhibit the xanthine oxidases—they intervene in the oxidative injury due to its reaction with molecular oxygen and then liberate the superoxide components. Generally, the phenolic compounds can induce antioxidant enzymes, for instance SOD, catalase, and glutathione peroxidase that break the superoxide anions, hydrogen peroxides, and hydroperoxides and then inhibit the generation of some enzymes, including the xanthine oxidases [23]. 

Nevertheless, the potential antioxidant activity of phenolic compounds is directly dependant on the category of the plant species used, the harvesting time, the growing conditions, the storage conditions as well as the type of the solvent used in the processes of extraction [91,100].

The polyphenols can exhibit antioxidant effects by various mechanism pathways to scavenge and inhibit the production of ROS [101]. Actually, previous studies have reported that polyphenol can react with the ROS by the donation of hydrogen atoms or electrons to the unpaired electrons to free radicals, and then generate the stability of phenolic oxygen radicals [58,101]. Consequently, these bioactive compounds can eliminate the free radical products [58]. Generally, the polyphenols, such as the tocopherols and flavonoids, are considered as considerable primary antioxidant compounds. Their aromatic amines can inhibit the autoxidation by the mechanisms of the electron transfer [101]. 

Furthermore, the polyphenols that are non-enzymatic antioxidants, for instance phenolic acids, ascorbic acids, simple phenols, and tocopherols, intervene to reduce the pro-oxidative reaction through the elimination of transition metal ions contaminants, scavenge the alkoxy and peroxy radicals, and generate the quenching of the oxygen in the singlet form [101].

### 3.2. Antihypertensive Activity

Hypertension often has no symptoms even although it is one of the main risk factors for cardiovascular diseases in the world. These disorders are considered among the most severe worldwide public health threats since they are the leading cause of death [102,103]. Although the causes of hypertension are not well known, aging can be a risk factor because most people with hypertension are 60 years old or older, and it is a major risk factor for heart disorders, myocardial infarction [102]. In fact, hypertension can exert a great effect on morbidity and mortality via the generation of various complications. Consequently, the prevention and treatment of hypertension have become essential for inhibiting their devastating complications. Dietary adjustment is considered an important regulation method for the modulation of hypertension. Moreover, the principal efficient non-pharmacological interventions are represented by weight loss, intensification of physical activities, reduction of sodium, diet, and supplementation of potassium [103]. The antihypertensive activities of some phenolics are reported in Table 3.

However, in order to treat hypertension, several synthetic and chemical drugs have been used and are recommended by the Word Health Organization (WHO), such as β receptor blockers, angiotensin II receptor antagonists (ARBs), calcium antagonists, angiotensin-transferase inhibitors, and diuretics [109]. In fact, the antihypertensive impact of these drugs is effective; however, they have several contractive and negative effects, such as vertigo, ankle swelling, sodium and water retention, cough as well as elevated blood lipids [109,110]. Moreover, the use of these drugs cannot alleviate the symptoms of hypertension and cannot be utilized for a long time to treat this arterial disease. 

The development of novel and non-toxic drugs as an alternative to synthetic ones for the prevention and treatment of hypertension becomes more indispensable. In this context, several research studies regarding the natural and phytochemical compounds have been established to discover promising therapy for hypertension disease. Accordingly, polyphenol compounds as secondary metabolites offer an opportunity to treat this disease. It has been reported that phenolic compounds, including phenolic acids, have several biological potentials and can act as antihypertensive agents.

Furthermore, the polyphenols as bioactive compounds are mainly found in different aromatic and medicinal plants as well as in many foods, such as tea, soybeans, fruits, and vegetables, which are characterized by their preservative impact on the development of hypertension and cardiovascular diseases [111].

Nevertheless, it has been reported that endothelial dysfunction is one of the hallmarks of hypertension disease. Thus, the endothelial dysfunction is characterized by the association of the endothelium-dependent vasodilatation with oxidative damage. In fact, this dysfunction is detected before the modifications in the structure or texture of the arterial walls and consequently this impairment in the function contributes to the beginning and the generation of cardiovascular diseases [112]. Previous studies have demonstrated that phenolic compounds and their derivatives can exert an advantageous impact on the vascular endothelial functions by some mechanisms, such as the normalization of the angiotensin system, the augmentation of the level of nitric oxide (NO), endothelium dependent hyperpolarization (EDH), and the prevention of oxidative stress through the inhibition of the appearance of pro-oxidant enzymes. For instance, the COXs and the NADPH oxidase can contribute to the improvement of endothelial function and the prevention of vascular longevity and, thus, to the protection against hypertension [112].

Furthermore, the polyphenols can induce anti-hypertensive activities via other mechanisms, including the activation of the AMPK pathways through the LKB1, the inhibition of the phosphorylation of the protein mammalian target of rapamycin (mTOR) and p70 ribosomal protein S6 kinase (p70S6k) as well as the increase of SIRT1 activation [113]. Moreover, the phenolic compounds can participate in the decrease of hypertension via the activation of the mTORC2-Rictor survival pathway as well as reduction of the expression of the mTOR signaling proteins [113].

Additionally, the role of polyphenols present in different sources (black currant, beet root, pomegranate, everlasting flower) in the prevention of endothelial dysfunction has been elucidated in several studies [114,115,116,117]. The polyphenols could prevent activation of the angiotensin system, enhance endothelial relaxation by regulating EDH, oxidative stress, and inflammatory response to improve the angiotensin system producing the vasodilation effect [112,114,116,118].

Furthermore, according to several experimental results, it has been reported that the proanthocyanidins as an important group of phenolic compounds have a vasorelaxant effect because of the considerable role of nitric oxide (NO). In fact, the polyphenols can protect the human umbilical vein endothelial cells and then contribute to the prevention of hypertension and its complications [119].

It has been reported in previous in vivo research studies that polyphenols and their derivatives have an important antihypertensive effect because of their contribution to the reduction of systolic blood pressure detected in the spontaneously hypertensive rats [120,121]. 

In addition, these bioactive compounds, which are found generally in herbal medicine, have been used clinically due to their advantages in the treatment of hypertension and being characterised by the reduction of the prevalence of complications related to metabolic abnormalities [109]. Moreover, it has been proved by scientific research that the flavonoids, which are considered the main active phenolic compounds in several medicinal plants, have considerable effect on the improvement of cardiovascular functions, and the prevention and the treatment of hypertension [122]. Therefore, the principal classes of flavonoids characterised by their antihypertensive impact are luteolin, chrystin, linarin, and apigenin [109]. It has been demonstrated that anthocyanin, as an important class of polyphenols, has a vasodilation activity. Their mechanism has been represented by the regulation of the activity of endothelial nitric oxide synthase (eNOS) in endothelial cells (ECs), inhibition of angiotensin-converting enzyme (ACE) activity, and the thromboxane pathway and the regulation of arterial blood pressure [109]. Moreover, quercetin, as a phenolic compound, can contribute to the reduction of the spontaneous hypertension impact by the enhancement of vasodilatation and blood viscosity [122].

Furthermore, polyphenols have an effective antihypertensive effect through several mechanisms of action. The antihypertensive treatment with polyphenols is manifested by the improvement of endothelial function as well as the relaxation of vascular tissues that appear through ACE inhibition and the nitric oxide-cyclic guanosine monophosphate (NO- cGMP) pathway [102]. Thus, the phenolic compounds can stimulate the endothelium-dependent vasodilation, and inhibit the synthesis of the vasoconstrictorendothelin-1 (ET-1) [102]. It has been shown that polyphenols can also contribute to the reduction of the activity of metalloproteinases, which have an important role in vascular dysfunction and the generation of many categories of cardiovascular diseases, including hypertension [102,123]. 

### 3.3. Immunomodulatory Activity

**Figure 3:**
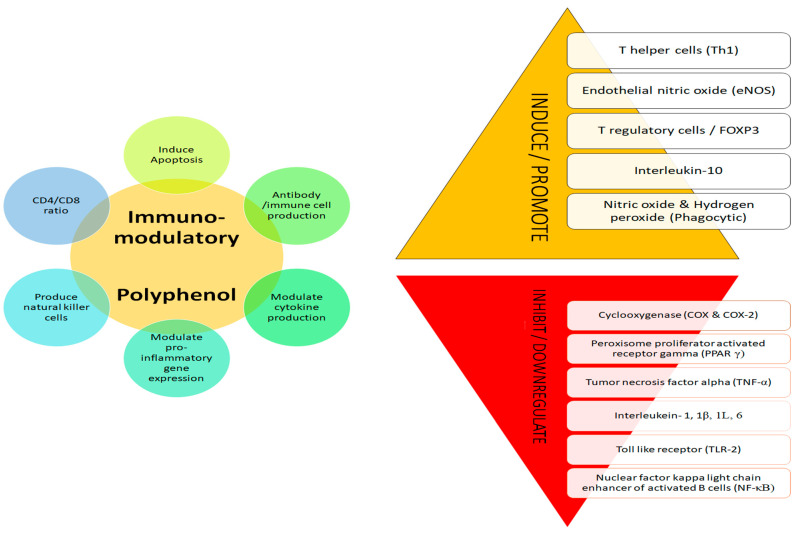
Immunomodulatory mechanism of polyphenol.

The immune system plays a vital role in well-being by increasing immune response and providing protection [124]. Polyphenols have well demonstrated immunomodulatory effects as they regulate the immune cells, macrophages, cytokines, signalling pathways and influence dendritic cells and lymphocytes (B and T), suppress T cell activation and natural killer cells, and suppress tumour-associated macrophages (Figure 3) [125,126,127,128,129,130]. High immunomodulatory impacts were associated with high antioxidative properties [6]. The negative impacts of synthetic drugs and the quest for natural alternatives for therapy has led to an increased demand for multi target action of phenols for enhancing immunity [127,130].

Cytokines and inflammation are the mechanisms used by the body to maintain homeostasis by eliminating harmful stimuli. Cytokine are known to mediate immunity by acting as pro-inflammatory (IL-2, IL-8, TNFα, IL-6, IL-8, IFN-ɣ) and anti-inflammatory (IL-4, IL-10, TGFβ) agents [129]. Moreover, different signalling pathways such as nuclear factor kappa-light-chain-enhancer of activated B cells (NFκB), mitogen-activated protein kinase (MAPK), and arachidonic acid signalling pathway have been reviewed as related to innate and adaptive immunity [129,131]. Polyphenols have been discussed extensively to impact cytokines, managing inflammation and modulating immunity [129,131]. Polyphenols have been reported to exhibit anti-inflammatory activity through different mechanisms such as by inhibiting pro-inflammatory molecules (TNF-α, IL-1β, IL-6, IL-8, iNOS, TLR2, TLR4), downregulating PKC-NFκB pathways inhibiting the production of pro-inflammatory molecules, and inducing production of Nrf2 signalling [132,133,134,135,136]. Furthermore, polyphenols are reviewed by Shakoor and others [137] to modulate the immune system by acting on dendritic cells (initiating immunity), macrophages (maintaining ratio between pro and anti-inflammatory activity), natural killer cells, T and B cells, and by T cell differentiation and regulation of inflammation.

Polyphenol-rich cranberry juice reduced the risk of infections by improved proliferation (Ɣδ-T) of cells, usually taken for reporting the improvement on immune function [138]. Improvements in urinary tract infection for patients using cranberry juice have been reported [11]. Another study showed that resveratrol was abundant in grapes and berries and regulated macrophages known for their ability to activate the immune system (TLR) against infection [139]. Bilberries rich in polyphenols were evaluated for their anti-inflammatory properties [140]. Diet supplementation with 400 g of fresh bilberries decreased the concentration of IL-2, IL-6, LPS, and CRP. The decrease in all cases was characterized by pro-inflammatory activities of bilberries decreasing inflammation.

Resveratrol was also known to irreversibly inhibit the production of cytokine (IFN-ɣ, IL-2, TNF-α) and signalling pathway (NF-κB) [141]. Resveratrol analogues inhibited the inflammatory response by inhibiting enzymes and pathways reducing cytokine mediated inflammation and improving immunity [142]. Curcumin has been reported to modulate inflammatory cytokines, inhibit enzymes (cyclooxygenase and lipoxygenase) responsible for inflammation, and lower the rate of signalling pathways [129]. The ability of curcumin analogue as an anti-inflammatory agent to inhibit NF-κB was evaluated by Olivera et al. [143]. Curcumin analogue (EF31) inhibited NF-κB ability to bind with DNA, nuclear translocation and induction, reducing pro-inflammatory effects. 

Tea, a major source of the polyphenolic compound catechins (epicatechin-EC, epicatechin gallate-ECG, epigallocatechin-EGC, and epigallocatechin gallate-EGCG), was evaluated for its ability to produce inflammatory cytokines [144]. EGC and EGCG (10 and 20 µM) inhibited the pro-inflammatory cytokines (IL-1β). On the contrary ECG, EGC, and EGCG (10 µM) increased the production of anti-inflammatory cytokines (IL-10). ECG, EGC, and EGCG have anti-inflammatory action when evaluated as ratio of IL-1β against IL-10. Cocoa polyphenol significantly reduced pro-inflammatory cytokines in activated THP-1 cells. Significant reductions in pro-inflammatory cytokines were also observed [145]. Tea catechin exhibited in vivo cytotoxic activity against natural killer cells [11].

Polyphenols present in pomegranate peel were evaluated for their anti-inflammatory activity through suppression of mitogen activated protein kinases (MAPKs) by Du et al. [146]. Findings highlighted strong anti-inflammatory activity of polyphenols from pomegranate peel and significant inhibition of pro-inflammatory cytokines (TNF-α, IL-1β, and IL-6) was observed. The anti-inflammatory impacts were attributed to inhibition of MAPK signalling pathways mediated through inducible nitric oxide synthase (iNOS) and cyclooxygenase-2 (COX-2) expression.

The phenolic compounds can modulate the immune functions via different mechanism pathways, including the inhibition proliferation of mononuclear cells from peripheral blood stimulated by mitogens. In addition, polyphenols can induce an immunomodulatory activity through the reduction of the molecule co-stimulatory, the prevention of the activation of MAPK, and also the translocation of nuclear factor-κB (NF-κB) [147]. These bioactive compounds can participate in the regulation of the activities of various transcription agents, for instance activator protein-1 (AP-1), NF-κB, signal transducer and activator of transcription (STAT) [147].

### 3.4. Antimicrobial Activity

**Figure 4:**
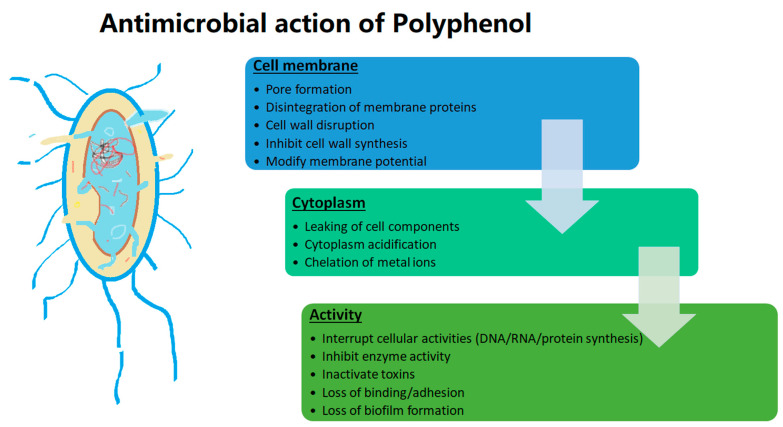
Antimicrobial activity of polyphenol.

Microorganisms are of critical concern for humans, as they are responsible for causing several infections and are accountable for several deaths in severe cases [148]. However, the usage of antimicrobials from synthetic sources and their excess usage have led to development of resistance against antimicrobials responsible for increased incidence and virulent strains [148,149]. Hence, the quest for alternate naturally occurring compounds showing antimicrobial activity has gained immense importance [150]. Polyphenols have gained specific importance due to their diverse mode of action against a large microbial populations, which is why they have an antimicrobial effect, and at the same time they benefit favourable microorganisms [149,151]. The antimicrobial activity of some polyphenols is tabulated in Table 4 and illustrated in Figure 4.

Polyphenols are known to exhibit antimicrobial action by disturbing the lipid membrane, altering the structure leaking cellular constituents and disturbing intracellular functioning, inhibiting/inactivating enzymes, impacting the degree of hydroxylation as well as level of oxidation [148,149,152]. Novel extraction and encapsulation technologies have been reviewed to enhance the antimicrobial activity of polyphenols [92,153,154]. 

Furthermore, polyphenols can generate antimicrobial activity so these bioactive compounds provoke an alteration of the permeability of the microbial cells, leading to the destruction of the cellular composition [101]. Moreover, the hydroxyl group (OH^−^) that characterises the phenolic compounds has a considerable role in the death of bacterial cells. Hence, the interaction of bacterial cell wall with “OH^−^” of polyphenols can produce the destabilization of proton interchange, reduce the gradient through the cytoplasmic membrane of bacterial cells, and decrease the ATP pool, and, thus, generate the death of microbial cells [101].

Phenolics from Japanese apricot (umeboshi) were evaluated for antimicrobial property against Enterobacteria [155]. Phenolics exhibited higher antimicrobial activity against evaluated strains but at relatively higher concentration (1250–5000 µg/mL). The chemical analysis revealed the presence of hydroxycinnamic acids and chlorogenic acid derivatives. *Salvia leriifolia* extracts were evaluated for antimicrobial activity [156]. The higher phenolic composition is related to high antimicrobial activity. The highest inhibition was observed for *Pseudomonas aeruginosa*, *Staphylococcus aureus,* and *Escherichia coli* (MIC-80, 110 and 120 mg/mL), respectively. Higher phenol present (TPC- 178 mg GAE/100 g) in *Pistacia atlantica* subsp. kurdica hulls essential oil (178 mg GAE/100 g) was attributed to the antimicrobial activity of essential oils derived from *Pistacia atlantica* subsp. kurdica hulls [157]. Higher control was observed in Gram positive bacteria over Gram negative bacteria due to their low susceptibility to polyphenols.

**Table 4:** Antimicrobial activity of phenolic compounds.

Source	Compound	Main Findings	Reference
Turmeric powder	Polyphenol	Grinding, i.e., reducing the particle size increased the antimicrobial activity. Polyphenol content also improved efficiency.	[158]
*Matricariaaurea*	Phenols	Gram positive bacteria were inhibited (MIC—0.4–12.5 mg/mL) and gram negative bacteria were found resistant (MIC—25–50 mg/mL).	[159]
Grape seed extract and pine bark extract	Gallic acid, vanillic acid, caffeic acid, ferullic acid	Inhibited *E*. *coli*, *Salmonella*, *L. monocytogenes*, and *A. hydrophila.*	[160]
Grapefruit seed extract	Naringin	Effective inhibition of pathogenic indicator organism was observed at lower concentration in comparison with positive control.	[161]
*Rumextingitanus* leaves extract	Total phenolics and flavonoids	Ethyl acetate extract inhibited gram positive (MIC—0.312–10 mg/mL) and pathogenic microorganisms.	[162]
American cranberry (*Vacciniummacrocarpon*) fruit pomace	Polyphenols (34%)––catechins, procatechuic acid, chlorogenic acid, epicatechin, trans-cinnamic acid	Extract (2–8 mg/mL) exhibited significant inhibition of 12 strains of *Listeria* strains. In meat, a model protein rich matrix had impact on antibacterial activity.	[163]
Arugula (*Erucasativa*) seeds extract	Flavonoids	Methanol extract inhibited *S*. *aureus* and *B*. *Cereus* (MIC- 80 µg/mL).	[164]
Olive leaf extract	Luteolin-7-*o*-Glucoside, Luteolin-4-*o*-Glucoside, Oleuropein, and Vabascoside	Complete inhibition of *L*. *monocytogenes* and *S*. *entertidis* and *E*. *coli* (95%) was obtained using 62.5 mg/mL extract. Biofilm formation of *L*. *monocytogenes* and *S*. *entertidis* was also inhibited.	[165]
Clove essential oil	Phenols	Encapsulation masked the strong odor of clove limiting application. In vitro inhibition of *S*. *aureus*, *E*. *coli,* and S. Typhimurium. High total phenolic composition (9.07 GAE mg/g).	[154]

Wine industry by-products (skins, stems, seeds, and skins) were evaluated for their antimicrobial potential due to the abundance in phenolic content (TPC- skin-360.2 µg/mg, seeds-363 µg/mg, stem-226.8 µg/mg) [166]. Results highlighted a direct relation between phenols and antimicrobial activity, and a significant reduction in the antibiotic resistant strains was reported. The highest antimicrobial activity was observed against *L*. *monocytogenes* (seeds), *E*. *faecium* (seeds), and *K*. *Pneumonia* (skins). Different plant parts of *Moringa oleifera* (leaf, root, flower, bark, and seed) extract (methanolic, ethanolic, ethyl acetate, water, and acetone) were evaluated for their antimicrobial potential [167]. Amongst the plant parts evaluated, moringa leaf (112 mg/g) and extract of ethyl acetate (200 mg/g) and aqueous (69 mg/g) extract had the highest levels of total phenolics and total flavonoid content, respectively. Myricetin was found in highest concentration, followed by quercetin, responsible for the antimicrobial activity. Ethanolic extract from leaves exhibited the highest inhibition of *P*. *aeruginosa* and *E*. *carotovora*. Similarly, a recent study highlighted the antimicrobial activity of ethanolic extracts from *Centellaasiatica* on six evaluated indicator organisms [168]. MIC ranged from 62.5–125 (25% ethanolic extract) to 7.81–125 (50% ethanolic extract). The results were related to higher total phenolic (69.54 mg GAE/g) and flavonoid (13.90 mg QE/g) composition present in the ethanolic extract.

Phenolic extracts (flavonoid) from *Lavandula stoechas* were evaluated against pathogenic microorganisms [169]. The phenolic extract exhibited antimicrobial activity against all evaluated strains (*E. coli*, *K*. *pneumonia*, *S*. *aureus*, *E*. *cloacae*, *A*. *baumanii*, *P*. *aeruginosa*). The minimum inhibitory concentration ranged from 10 to 40 mg/mL.

Furthermore, due to high antimicrobial activity and diverse mechanisms, polyphenols are under wide application to treat several infectious diseases. A recent study by De Angelis and others in 2021 [170] reported the antiviral impact of polydatin (precursor of resveratrol) against influenza virus. The application significantly lowered MTT assay on vero E6 cells at a concentration of 80 and 100 µg/mL. Similarly, application inhibited replication of the virus for the 24 h duration observed and viral titre from supernatant of infected treated cells [170]. Moreover, several reviews focused on the specialized ability of polyphenols to protect against human diseases from noncommunicable, viral diseases to oral microorganisms are recently reported [171,172,173,174,175,176].

### 3.5. Anticancer Activity

Nowadays, cancer is one of the major chronic diseases occurring in the modern world [177]. Cancer generation is mainly associated with the uncontrollable development of tumor cells. Thus, this disease has received considerable attentions worldwide. The WHO has reported that many people around the world are affected by this disease every year [178]. In fact, global statistics indicate that there were about 14.1 million new cases of cancer and about 8.2 million deaths worldwide in 2012 [177]. As a result, cancer has remained the disease most frequently responsible for the deaths of many people around the world.

The results of epidemiologic studies have suggested that the etiology of cancer is principally attributed to the variations in lifestyles, ever-increasing urbanization, the predominant diet, and successive changes in environmental conditions [177,178]. 

Although the traditional treatment of cancer, such as chemotherapy, radiotherapy, immunotherapy, and surgery have a considerable impact on the therapy of this chronic disorder, the generation of cases of toxicities, the apparition of drug resistances, and the high costs of therapy represent the main problems to treat cancer patients [179]. Therefore, it is important to develop effective and non-toxic antitumor drugs derived from natural product resources, which have become an area of research. Anticancer activity of some polyphenols is tabulated in Table 5.

Plant polyphenols are one of the most widespread bioactive compounds. They are characterised by high structural varieties which, in turn, generate several ranges of biological properties, including anticancer effectiveness [177]. Recently, polyphenols have received considerable attention for cancer treatments because they have fewer side effects and low toxicity. In this context, epidemiological, preclinical, and clinical research have shown that the daily consumption of polyphenols has a strong correlation with the prevention of different types of cancer [179]. Several data indicate that polyphenols have numerous target actions, and they can transmit multiple cell signaling pathways to exert their anticancer effects against different types of cancers. In fact, many investigations have revealed that polyphenols can exercise their anticancer impacts by regulating numerous cell signaling pathways.

Moreover, these compounds regulate the activity of certain enzymes and other useful proteins [180]. The phenolic compounds can, thus, affect the carcinogenesis process through several mechanisms, making their use appropriate to treat different varieties of cancer [177]. Nevertheless, the principal obstacles to successful treatment based on these bioactive compounds are their metabolic modifications, weak membrane permeability, low systemic bioavailability, physiological fluctuation, and oxidative damage [181,182]. Therefore, the bioavailability of polyphenol compounds has been identified as the proportions of the substance reaching the circular system and distributed in numerous tissues [4]. In fact, several studies on enhancement of polyphenol bioavailability are ongoing to overcome difficulties in reaching their therapeutic concentrations in target tissues [177]. Moreover, the bioavailability and biotransformation appear to be the two causal characteristics that affect the effectiveness of polyphenols. Based on the dimensions, polyphenols can be absorbed by small and/or unimpeded intestinal interfaces [4]. Thus, it has been suggested that polyphenols are common in conjugated form in plasma. However, it has been demonstrated that conjugation with proteins in the oral cavity and the acid pH of the stomach does not alter the stability and biological activity of polyphenols.

However, the anti-cancer characteristics demonstrated are primarily attributed to their anti-inflammatory, cell cycle-stopping, anti-metastatic, anti-angiogenic, autophagic, anti-proliferative, and apoptotic effects [178]. Nevertheless, the regular consumption and utilization of dietary phytochemicals, which are generally present in plant-based foods (such as vegetables, fruits, tea, and cereals) and used as food additives, may be a promising significant approach in the prevention of cancer disease [178,183,184]. Thus, many research studies have demonstrated that the dietary polyphenols have an important role in the prevention of cancer.

Resveratrol (stilbenes), anthocyanins, curcumin, and epigallocatechin-3-gallate (EGCG) are considered as polyphenols compounds, which are secreted and produced in response to environmental stimulators such as stress. These compounds are characterised by their protective efficiencies in some cancer models by various signaling pathways [42,185,186].

Moreover, phenolic molecules are considered the main health-protective bio-compounds, such as proanthocyanidins, flavonoids, and hydroxycinnamates, which possess anticancer properties and, thus, reveal their potential therapeutic and chemo-preventive effects [187].

Furthermore, several preclinical studies have investigated the anticancer effects of resveratrol and have demonstrated that this compound has a considerable prevention impacts against different types of cancers including digestive tract, breast, skin, lung, and prostate cancers [4]. Moreover, previous research has reported that the synergic impacts of the polyphenol compounds of catechin, resveratrol, and quercetin are more important than their individual effect on the inhibition of breast cancer cell progression, cell cycle proliferation, and primary mammary tumors [188].

**Table 5:** The anticancer properties of some phenolic compounds.

Sources	Compounds	Assays	Main Findings	Reference
- Green tea	- Epigallocatechin-3-gallate (EGCG)	- In vitro assay: B16-F3m melanoma cells. - In vivo assay: injection of B16-F3m melanoma cells into Balb/c mice.	In vitro: EGCG can inhibit the migration of B16-F3m cells as well as their invasion. Moreover, there is inhibition by the EGCG of the homotypic cell aggregation and also the activity of MMP-9 (matrix metalloproteinase-9) as well as the tyrosine phosphorylation of focal adhesion kinase (FAK). In vivo: EGCG can decrease lung metastases in mice bearing B16-F3m melanomas but it can increase the survival rate of melanoma-bearing mice.	[189]
- *Derris eriocarpa*	- Flavonoids: Alpinumisoflavone (AIF)	- In vitro assay: SK-MEL-1 and A375 human melanoma cells. - In vivo assay: AIF was used to treat xenograft mice by intragastric administration.	In vitro: the use of AIF can contribute to the inhibition of the migration, invasion, and proliferation of tumors. In vivo: the AIF participates in the inhibition of mRNA expressions of MMP-2, MMP-9 as well as the inhibition of lung metastasis.	[190]
- Purified flavonoid compound	- Flavonoids ✓ Apigenin ✓ Quercetin	- In vitro assay: The use of B16-BL6 murine melanoma metastatic cells. - In vivo assay: the injection of phenolic compounds to B57BL/6N mice and the evaluation of their potential activity on the inhibition of melanoma lung metastasis.	In vitro: the use of phenolic compounds, apigenin and/or quercetin, can inhibit the TNF-α-induced VCAM-1 expression and decrease the adhesion of melanoma cells to lung sections. In vivo: inhibition of lung metastasis and the melanoma cell adhesion to vascular lung endothelium.	[191]
- Rhizome of *Curcuma longa* L.	- Curcumin	- In vitro assay: B16-F10 murine melanoma metastatic cells. - In vivo assay: the injection of curcumin into C57BL6 mice.	In vitro: the treatment with curcumin can increase the tumor-suppressor genes tissue inhibitor metalloproteinase (TIMP-2) as well as the expression of E-cadherin and nonmetastatic gene 23 (Nm23). Moreover, this bioactive compound can contribute to the decrease of the binding of the treated cells to 4 extracellular matrix (ECM) proteins. Furthermore, there is a reduction of the binding to vitronectin, fibronectin, and collagen IV. Moreover, decrease in the expression of α5β1 and α (v) β3 integrin receptors. In vivo: the curcumin can contribute to the decrease of lung metastasis.	[192]
- *Camellia sinensis*	- Catechin nanoformulation	- In vitro assay: WM266-4 human melanoma cells. - In vivo assay: the xenotransplant of WM266 human cancer cells in zebrafish embryos.	- In vitro: decrease of the proliferation of cells as well as their mobility and the increase of cell death and cell doubling time. - In vivo: inhibition of tumor neo-angiogenesis.	[193]

In contrast, it has been revealed that polyphenol compounds can contribute to the treatment of cancer disease by various biological processes. Therefore, the cancer prevention and regulation by these bioactive compounds have targeted the expression of genes, cell cycle proliferation, development, migration, and progression of cells. In addition, the cytoprotective and anticancer properties of the polyphenol substances can be generally attributed to their pro-oxidant and antioxidant attributes [4,194]. In this regard, the anticancer activity of polyphenols can be manifested by various mechanisms. Thus, these bioactive compounds have demonstrated important anticancer properties due to their role in the scavenging of ROS and other free radicals, which is manifested by the transfer of the electrons to the oxidants. Consequently, the strong antioxidant activities of polyphenols lead to a decrease of the mutation and damage of DNA, which can cause genetic diseases and cancer. Thus, the polyphenols lead to the generation of suppression of the cell cycle, apoptosis, down-regulation of proliferation of cells via different modulations of many signaling pathways, such as phosphatidylinositide 3-kinases/protein kinase B (PI3K/Akt), epidermal development factor receptor/mitogen activated protein kinase (EGFR/MAPK), anti-inflammatory factors, and nuclear factor kappa-light-chain-enhancer of stimulated B cells (NF-kB) [4].

Polyphenols can exhibit anti-cancer effects via other different mechanism pathways, for instance the perforin-granzyme apoptotic pathway, mitochondrial-mediated apoptosis by over generation of ROS, and the death receptor pathway [195,196,197]. Moreover, the phenolic compounds can induce the regulation of metabolism, development of the cell cycle, and the inhibition of tumor expression through the p53 mechanism pathway [186]. Furthermore, these compounds can stop the replication of DNA, the transcription of RNA, and repair the damage of DNA of the cancer cells [198,199,200].

## 4. Antiviral Activity of Polyphenols against COVID-19

The COVID-19 pandemic was caused by SARS-CoV-2, with 386,548,962 confirmed cases of infection and 5,705,754 deaths reported by January 2022 [201]. The envelope/structural protein protection around the coronavirus makes antiviral action difficult. Further, the mutation in SARS-CoV-2 (omicron) has become a variant of concern due to increased transmissibility and ability to escape the immune system, and has posed a risk of another community spread [202]. Considering the replication occurring in viruses, the therapy targeting virus receptors and improving immunity are required [203]. Owing to the spread of the pandemic, the role of nutraceuticals and functional foods has gained special importance [204]. The well-known ability of polyphenols to enhance immunity has gained special attention in this pandemic and several studies have highlighted the ability of phenols against SARS-CoV-2 [171,204,205].

Bahun et al. [206] reported SARS-CoV-2 inhibition (in-vitro) by plant polyphenols. Amongst the evaluated quercetin (23.4 µM), resveratrol (16.9 µM), epigallocatechin gallate (13.9 µM), curcumin (11.9 µM), and ellagic acid (11.8 µM) exhibited the highest inhibition levels (IC_50_). The inhibitions were linked to polyphenols’ stable binding with active site formation of hydrogen bonds, forming hydrophobic interactions. Polyphenols could strongly bind with SARS-CoV-2 receptors, preventing entry in the host, PLpro/3CL^pro^ substrate, regulate ACE 2 expression and functioning, and inhibit protease [171,203,207,208,209,210].

Phenolic compounds were evaluated for their SARS-CoV-2 inhibitory activity by Xiao et al. [211]. Amongst the evaluated compounds, myricetin exhibited the highest inhibition of SARS-CoV-2 (IC_50_- 3.684 µM). The higher level of viral inhibition was attributed to binding with 2 M^pro^ and forming hydrogen bonds. Additionally, myricetin effectively reduces inflammation in lungs [211]. In-vitro inhibition of SARS-CoV-2 cells by resveratrol (4.48 µM) was reported [212]. The effect was attributed to the generation of nitric oxide, which further helps in relieving inflammation. Naturally occurring polyphenols kamferol (−7.4 kcal/mol), quercetin (−8.5 kcal/mol), and fisetin (−8.5 kcal/mol) showed binding and interaction affinity with spike protein [213]. Flavonoids inhibited 3CL proteases/enzymatic activity of SARS-CoV-2. Herbacetin (−9.263), rhoifolin (−9.565), and pectolinarin (−8.054) exhibited binding affinities confirming the inhibition of enzymes enhancing the antiviral activity. 

## 5. Conclusions and Future Prospect

The toxicity and the undesirable side impacts of synthetic and chemical molecules and the resistance of some microbial species to chemical drugs lead several scientific researchers to discover other alternative sources of bioactive compounds. Thus, there is an increasing demand for products of natural origin such as the polyphenol compounds, which constitute a single group of phytochemicals present in vegetables, fruits, herbs, and other natural sources. 

The current review has shown that the phenolic compounds constitute a highly multifunctional and diversified group of bioactive compounds with numerous beneficial impacts in many areas. In fact, these compounds have been associated with human health. In this regard, there is a growing interest in their potential application in food industries as well as in therapeutic and pharmacological sectors. Their activity is supported by their functional groups, which are capable of accepting the negative load of a free radical. This review has also summarized the positives effects and biological activities of polyphenols and their significance for human health due to antioxidant, antimicrobial, anti-hypertensive, anticancer, immunomodulatory, and antiviral properties. Therefore, the application of polyphenols, such as flavonoids, catechin, tannins, phenolic acids, etc., in the food industry as bio-preservative substances for foods and beverages can lead to a superb activity on the inhibition of oxidative stress by different types of mechanisms. Additionally, these compounds can reduce the lipid oxidation in the human body, prevent the organs and cell structure against deterioration, and protect their functional integrity, since the polyphenols have strong antioxidant properties. 

Moreover, the antioxidant features of polyphenols can contribute to the preservation and protection of human health against diverse diseases, in which oxidative damages are involved as a contributing and casual factor. There are a several investigation studies regarding the health benefits of polyphenol on hypertensive and cardiovascular diseases. In this context, these investigations have included lipoprotein oxidation, which is related to hypertension through endothelial dysfunction and then contribute to cardiovascular disease production. Furthermore, the antioxidant properties of polyphenols can decrease the generation of ROS and the reduction of inducible nitric oxide synthase (iNOS) expression. Consequently, these properties contribute to the inhibition of DNA damage, metastasis blockage, inhibition of cell cycle progression, and stimulation of apoptosis of malignant cells. All of these make polyphenols promising compounds that can be used for the treatment and prevention of cancer diseases. Thus, polyphenols have superb anticancer and antitumor properties because of their immunomodulatory effects on different type of cancer cells. Thus, they can regulate the production of chemokine and cytokine as well as activate the immune cells. 

Additionally, this review has reported that polyphenol compounds have important antimicrobial properties and are characterised by their potential to substitute synthetic chemical antibiotics. Further, bioactive compounds derived from polyphenols can be applied as a good antiviral agent.

Despite the data relating to the properties of polyphenols, further efforts are needed to discover their exact mechanism of action, antagonism, synergism, and other interrelations among them, and how their appropriateness can be applied. Furthermore, clinical trials of these compounds are essential in order to develop efficient and safe alternatives to protect human health.

## Figures and Tables

**Table 3:** The antihypertensive properties of some phenolic compounds.

Source	Compound	Assays Used for the Evaluation of Hypertensive	Main Findings	Reference
Purified compounds. Bitter orange, lemon, cocoa, and grapefruit.	- Apigenin - Diosmin - Other flavonoids	- Angiotensin-converting enzyme activity. - Vascular function.	The results of this research work demonstrated that the flavonoids used in this study have excellent antihypertensive effects and can be used as functional food agents due to their therapeutic role for arterial hypertension.	[104]
Purified flavonoid compound	- Quercetin	- Baroreflex sensitivity (BRS). - Heart rate (HR). - Arterial pressure (MAP).	The treatment of spontaneously hypertensive rats (SHR) with quercetin can decrease their hypertension and then enhance the BHR through the inhibition of oxidative stress.	[105]
Spanish red wines	- Kaempferol - Rutin - Myricetin	- Vasodilatory properties. - Aortic rings.	The findings of this research study demonstrated that there is an excellent correlation between the level of polyphenol compounds (especially the kaempferol) and the vasodilatory impact, which contribute to the prevention of hypertension and cardiovascular disease.	[106]
Purified flavonoid compound	- Rutin	- Cardiovascular functional modifications. - Two-kidney one-clip (2K1C) method. - Estimation of plasma renin content.	The antihypertensive impact of rutin as a bioactive compound can contribute to the regulation of hypertension due to its ability to scavenge free radicals, inhibit lipid peroxidation, and inhibit the plasma renin inhibitory effect.	[107]
*Phoenix sylvestris* (L.)	- Flavonoids - Tannins.	- Angiotensin-converting enzyme assay.	The phenolic compounds exhibited an excellent antihypertensive activity via ACE inhibition.	[108]

## Data Availability

Not applicable.
